# Green Thermal Aggregates: Influence of the Physical Properties of Recycled Aggregates with Phase Change Materials

**DOI:** 10.3390/ma16186267

**Published:** 2023-09-18

**Authors:** Zhiyou Jia, José Aguiar, Sandra Cunha, Carlos de Jesus

**Affiliations:** Centre for Territory, Environment and Construction (CTAC), Department of Civil Engineering, University of Minho, Campus de Azurém, 4800-058 Guimarães, Portugal; pg39237@uminho.pt (Z.J.); sandracunha@civil.uminho.pt (S.C.); cjesus@civil.uminho.pt (C.d.J.)

**Keywords:** recycled aggregate, physical properties of recycled aggregate, phase change materials, form stabilization

## Abstract

Increasing construction and demolition waste (CDW) and the large amount of energy consumption in the building operation process are high-profile issues at present. In the construction industry, recycled aggregated (RA) from CDW can be reutilized in construction, along with green materials, for example, as a road base layer, as aggregate in concrete, etc. Phase change materials (PCM) are often used as building materials due to their good latent heat storage properties. With the use of RA as a matrix to absorb PCM, a thermal performance aggregate can be obtained. This work studied the physical properties of RA from Portugal and combined PCM with RA to prepare a green thermal aggregate through two methodologies using a vacuum and atmospheric pressure. The green aggregate was used in concrete to observe its effect on the compressive strength of concrete. The results showed that the amount of PCM absorbed by the RA mainly depends on the porosity of the matrix material. At the same time, the volume expansion coefficient of PCM was 2.7%, which was not enough to destroy the RA. Ultimately, as the amount of green thermal aggregate increases, the compressive strength of concrete decreases. Green thermal aggregate prepared under vacuum conditions has a greater negative impact on the compressive strength of concrete.

## 1. Introduction

With the development of urbanization and large-scale building renovation activities, a large amount of construction and demolition waste (CDW) is produced. China, the USA, and the European Union (28 EU countries) are the countries and regions with the highest annual production of CDW in the world. The production quantities in these areas reached around 2.3 billion tons/year [1], 600 million tons in 2018 [2], and 807 million tons in 2020 [3], respectively. The composition of CDW can vary but typically includes materials such as concrete, metals, masonry, rock, asphalt, wood, sand, plastics, plasterboard, cardboard, soil, etc. [4]. These wastes not only occupy arable land but also have significant impacts on the environment, such as sedimentation, soil erosion, and increased greenhouse gas emissions [5]. Furthermore, it is important to note that the annual consumption of concrete, as a widely utilized building material, amounts to approximately 14 billion cubic meters worldwide. This massive consumption of concrete inevitably leads to a significant demand for natural aggregates [6]. Therefore, scientifically classifying and utilizing construction waste based on its material properties is an extremely urgent measure, which allows the producers to reduce negative impacts, achieves a circular economy, and promotes sustainable development in the construction industry [7].

In fact, CDW is a ubiquitous presence throughout all stages of construction, including the construction phase, decoration process, and demolition process. The composition of CDW is complex, among which solid waste, which is mainly composed of brick, stone, and concrete, is separated and treated to form CDW, commonly known as recycled aggregate (RA). This type of RA accounts for most CDW in many countries and regions [2,3]. Bravo et al. [8] reported that RA is generated from various building materials used in construction, renovation, and demolition activities; they researched the commercial fine RA found in the cities of Leiria, Sintra, and Faro in southern Portugal, and obtained the main components of RA. About 80–90% is of concrete, concrete products, mortar, non-bonded aggregates, natural stone, aggregates treated with hydraulic binders, and masonry, and about 10–20% are of bituminous materials, glass, and other materials. This also shows that the current recycled aggregate composition in Portugal is basically similar. In addition, the complexity of its constituent materials also makes it difficult for RA to achieve standardization in application.

The application of RA has received much research attention in the last century and it has been successfully used in road base layers, concrete, tiles, bricks, and so on [9,10,11]. Brito et al. [12] analyzed the volume index of this recycled ceramic aggregate (consisting of roof tiles, hollow bricks, and partition walls) and found that a small fraction of this recycled ceramic aggregate is nearly spherical. The water absorption rate of this recycled ceramic aggregate is 12%, which value is far greater than the water absorption of natural aggregates such as granite. The mechanical results showed that the strength of concrete with recycled ceramic aggregate is better than that of concrete with limestone aggregate. Bravo et al. [8] reported that the absorption percentage of water in recycled aggregate from Portugal is more than 10%. The results showed that with the incorporation of recycled aggregate at ratios higher than 25%, the mechanical properties decreased between 2% and 5% for each 10% increase in the recycled aggregate percentage.

Therefore, at the construction waste recycling station, a systematic process is employed to separate undesirable elements such as iron, soil, wood, and plastic from the initial CDW. Through meticulous manual selection, the recycling operators ensure that relatively pure recycled aggregates (RA) are obtained. To achieve the desired specifications, the RA is subsequently crushed and carefully sieved, allowing for the attainment of the required particle size. This logical sequence of steps ensures the production of high-quality RA suitable for various construction applications. Although the classification is relatively clear, the random matrix distribution of the composition of RA leads to some restrictions regarding the standardization of the use of these materials in concrete, especially in standards dictating the maximum volume of RA in concrete [13]. From this point of view, it is of great significance to strengthen our understanding of the main constituent materials of RA and each constituent material to optimize the standardized application of RA in concrete.

With the rapid growth of the global population and economic development, non-renewable energy sources such as fossil energy are increasingly being exhausted, and the energy crisis is becoming more and more serious, which will not only bring environmental problems but also cause a series of social security problems [14]. According to the “2020 Global Construction Industry Status Report” [15], building operations account for nearly 55% of global electricity consumption, accounting for about 20% of global total energy consumption. Therefore, reducing building energy consumption and improving energy consumption efficiency is critical. The report also pointed out that the building operation process not only consumed too much energy but also emitted about 10 billion tons of CO_2_, accounting for about 28% of the world’s total emissions. Facing the environmental challenges brought about by the burning of fossil energy, more and more researchers have paid attention to solar energy, a natural and harmless energy source. Through the performance of the phase change materials (PCM) themselves (when absorbing or releasing heat, the state changes, and the temperature remains unchanged), solar energy is applied to building maintenance components to achieve a natural constant indoor temperature state, which can greatly reduce building energy consumption [14,16]. 

There are four methods for applying PCM to building materials, such as direct incorporation [16,17], encapsulation [18,19,20], immersion [21,22], and form-stable methods [22,23,24]. Many researchers have presented studies in which they utilize PCM in construction products [24,25,26], namely, interior coating mortars, with the aim of increasing the thermal comfort of building occupants [27,28,29,30,31]. Cunha et al. [32] directly mixed pure paraffin into mortar to improve the thermal performance of the mortar and reduced the indoor temperature difference by about 3 °C in summer. 

The “form-stable composite PCM” method [23,24] consists of absorbing PCM in aggregates or frames with a certain porosity and then using them in building materials. For this method, the final thermal properties of the materials are determined by the amount of PCM absorbed by the aggregate, while the porosity/water absorption of the aggregate determines the amount of PCM that it absorbs. Shen et al. [22] studied the use of clastic light shale ceramist (CLSC) with a density of 300–500 kg/m^3^, a specific surface area of 2900 m^2^/m^3^, and a porosity of 58% to absorb paraffin for lightweight concrete. Finally, the CLSC was saturated with a 25% paraffin mixture, after which the thermal properties of concrete increased by 12.54%, 31.6%, and 41.23% when the paraffin content was 2% (30 kg/m^3^), 4% (60 kg/m^3^), and 6% (90 kg/m^3^) of the total concrete mass, respectively. Ryms et al. [33] studied expanded clay aggregate with a density of 1380 kg/m^3^, a diameter of 4–12 mm, and a water absorption of 18%, regarding the absorption of PCM. The results showed that the maximum possible amount of mixed PCM in concrete was 276 kg/m^3^. Zhang et al. [34] studied vermiculite as a supporting material to absorb PCM in the form of a lauric acid–palmitic acid–SA eutectic mixture, using the vacuum impregnation method. The results showed that the LA-PA-SA/VMT composite could reach a mass fraction of 50 wt% without any leakage of melted PCM. Lv et al. [35] reviewed the preparation, characterization, and application of nine kinds of clay mineral-based form-stable phase change materials (FSPCM), namely, kaolin, diatomite, sepiolite, montmorillonite, perlite, SiO_2_, attapulgite, vermiculite, and fly ash. The results showed that the preparation of FSPCM by the vacuum method is commonly used and that the thermal performance of the performance-oriented aggregate was improved. Zhang et al. [36] investigated the use of 0%, 1.83%, 3.62%, and 5.27% of carbon nanotubes as a filler in paraffin/expanded perlite form-stable phase change materials to enhance their thermal conductivity. When composed of 5.27% carbon nanotubes, the thermal conductivity of the materials was improved by 4.82 times more than materials with no carbon nanotubes. Frigione et al. [37,38] developed a thermal aggregate for use in mortar using Lecce stone (LS). The LS had a density of 2957 kg/m^3^, a porosity accessible to water of 33.6%, and particle sizes ranging from 1.6 to 2.0 mm. Polyethylene glycol (PEG), a PCM, was impregnated into LS through the vacuum impregnation method to create a form-stable composite PCM called LS/PEG. The LS/PEG composite achieved a PCM absorption rate of 23% by weight. Sarcinella et al. [39] further investigated the thermal properties of mortars incorporating LS/PEG. The results showed reduced cooling needs during the summer season and decreased heating requirements in spring. The cement-based mortar containing LS/PEG demonstrated the best thermal performance during four seasons. Costa et al. [40] devised a microstructure for a diatomite–vermiculite paraffin-based form-stable phase change material (PCM) employing the direct impregnation method, with the aim of enhancing the thermal performance of cementitious mortars. The findings revealed that the mortar exhibited the most favorable thermal behavior when it incorporated a 25% paraffin mixture. Specifically, the solid–solid and solid–liquid phase-transition temperatures were determined to be 49.33 °C and 57.29 °C, respectively, while the latent heat was measured to be 159.42 J/g. Uthaichotirat et al. [41] investigated recycled lightweight aggregate obtained from the manufacturing process of aerated concrete blocks, with a density of 374 kg/m^3^ and a percentage absorption of 67.5%, in terms of the absorption of Paraffin6035 (a type of PCM). This recycled aggregate with PCM presented a density of 566 kg/m^3^. Lightweight concretes were prepared with 25%, 50%, 75%, and 100% recycled aggregate, with PCM replacing the recycled aggregate. The results showed that the mechanical performance and thermal performance of concrete were enhanced with an increasing percentage of recycled aggregate with PCM content. 

The objective of this study is to develop a form-stable composite phase change material (PCM) by utilizing recycled aggregate (RA) as a supportive material and pure paraffin (PP) as the PCM component. The physical properties of RA and PP were characterized, such as the particle size distribution, density, and water absorption characteristics of the RA and the volume variation of PP, then two methods were selected to prepare green thermal aggregates, namely, vacuum-assisted and normal atmospheric pressure techniques. Subsequently, the absorption capacity of the RA for PP under normal atmospheric pressure and vacuum conditions was quantified, and the influence of the RA’s physical properties on PP absorption was analyzed. Additionally, the effect of green thermal aggregate, made using different methods, on the compressive strength of concrete was analyzed. The findings from this study will contribute to the development of advanced PCM-based materials with enhanced thermal performance and their distribution for sustainable and efficient energy storage applications.

The importance of this study is mainly reflected in the following. From the perspective of mitigating the consumption of natural raw materials in concrete production, the utilization of recycled aggregates holds paramount significance. On the other hand, the functionalization of reclaimed recycled concrete debris (RCD) aggregates and phase change materials (PCMs) has the potential to enable a scenario where the exclusive utilization of solar energy facilitates the reduction of energy consumption in buildings over their lifecycle.

Consequently, a discourse within this domain of research is becoming imperative as this field is poised to yield affirmative implications for the United Nations’ 2030 Agenda. Specifically, the following Sustainable Development Goals (SDG) will be positively impacted:

- SDG 1: End poverty in all its forms everywhere.
- SDG 7: Ensure access to affordable, reliable, sustainable, and modern energy for all.
- SDG 12: Ensure sustainable consumption and production patterns.

The innovation of this study can be summarized in the following points:

- Characterization of a recycled aggregate from Portugal.
- Functionalization of a recycled aggregate with PCM.
- Evaluation of two methods of incorporating PCM into the aggregate (under vacuum conditions and at atmospheric pressure).
- Evaluation of the mechanical properties of the concretes with the incorporation of RA functionalized with PCM.

## 2. Materials and Methods

### 2.1. Recycled Aggregate (RA)

The RA studied in this research was provided by a specialized CDW treatment company located in Figueira da Foz, situated in central Portugal. The aggregate is a commercial RA with a dimension of 0–10 mm. After screening, RA with a size of 4–10 mm was selected as the research object of this work.

Through careful observation and the analysis of construction waste recycling stations, it has been determined that the primary constituent materials and their respective sources are as follows: mortar waste mainly comes from the bond coat of bricks in the wall, while brick waste is primarily a constituent material of non-load-bearing and partially load-bearing walls in structures. Ceramics waste mainly comes from exterior wall tiles and indoor floor and bathroom wall tiles. Concrete waste comes from elements such as slabs and structural columns. Lime paste waste mainly comes from the insulation layer or leveling layer on the wall surface, and natural stone waste comes from the building’s facade or foundation structures. These materials are the most common types of CDW and can be recycled and reused in various applications. It should be noted that “plastic” waste is not pure plastic but is instead mixed with partially or completely wrapped stone or ceramic particles, exhibiting a mixed state. Additionally, there is a risk of broken soil particles soaking in water for a long time, although the proportion is not significant enough to impact the whole. The composition of glass is mainly transparent glass particles, although there may be some impurities, such as sawdust and so on.

### 2.2. Pure Paraffin (PP)

Paraffin is a combination of solid higher alkanes, consisting of a mixture of straight-chain alkanes (CH_3_–(CH_2_)–CH_3_). The CH_3_ chain manufacturing process releases a great deal of heat energy. For paraffins, increasing the chain length generally results in an increase in their heat storage capacity (enthalpy) and transition temperature [42]. Therefore, the paraffin transition temperature is directly related to the number of carbon atoms. Typically, paraffins have a transition temperature ranging from −12 °C to 71 °C and may exhibit an enthalpy between 128 kJ/kg and 257 kJ/kg [43]. The paraffin also has a high thermal energy storage capacity; heat storage and release occur at a relatively constant temperature, with no supercooling effect; it is chemically inert, has a long product life through phase change cycles, and the melting temperature ranges between −9 °C and 100 °C.

Pure paraffin (PP), commercially known as RT22HC and manufactured by the RUBITHERM^®^ company (Berlin, Germany), was chosen for this work as a thermal performance material. PP utilizes the phase transition process between solid and liquid states (melting and solidifying) to store and release thermal energy at a nearly constant temperature. The selected PP presented a temperature transition from the melting and solidifying ranges between 20 °C and 23 °C, with a main peak of 22 °C (Table 1) [44].

In Table 1, it is possible to observe the density, heat storage capacity, specific heat capacity, heat conductivity, flash point, and maximum temperature operation of the selected PP [44].

## 3. Experimental Program

### 3.1. Physical Characterization of RA

#### 3.1.1. The Proportion of Constituent Materials of RA

The proportion of RA constituent materials was determined by manual screening of the samples. Initially, the original RA was fully mixed, and the resulting mixture was subjected to a 24-h drying period in an oven at 105 °C. Subsequently, three samples, each weighing approximately 600 g, were randomly selected from the dried RA. Through careful classification and identification, the different constituent materials present in each sample were discerned, encompassing the complete range of materials composing the RA. Finally, the percentages of each material within the samples were calculated to ascertain their respective contributions to the overall composition of RA.

#### 3.1.2. The Particle Size Distribution of Aggregates

Analyzing the distribution of aggregates is important for ensuring the proper design and optimization of concrete mixes for specific applications. At the same time, the different particle size distribution will also affect the water absorption and density of the materials. Working according to the standard EN 933-1 [45], the particle distribution of the RA with a dimension of 4–10 mm was determined. Furthermore, after completing the procedures outlined in Section 3.1.1, the particle distribution determination was carried out for each material separately.

#### 3.1.3. Density and Water Absorption of Materials

Working according to the standard NP 581 [46], the density and water absorption of RA were tested. The experimentations were conducted in laboratory conditions at room temperature. A specific quantity of the sample was subjected to desiccation at a temperature of 105 °C for a duration of 24 h, denoted as M1. Subsequently, the sample was fully submerged in water for another 24-h period, allowing it to attain a state of saturation. The weight of the sample when saturated was recorded as M2. Further steps involved immersing the sample in water and suspending the scale at a measured distance above the water. The sample’s immersed weight was then determined and recorded as M3. The following calculations were performed using Equations (1)–(3) to determine the values of the waterproof material density, saturated density, and dry density of aggregates, respectively. Additionally, the degree of water absorption by the sample was evaluated utilizing Equation (4). 

This assessment holds significant importance for the subsequent experiment, involving the absorption of PP by the RA as a reference. Similarly, upon completion of the work in Section 3.1.2, the density and water absorption properties were determined for each material individually.

(1) $$Waterproof\;material\;density\;(kg/m^{3})=\frac{m1}{m1-m3}\times \rho$$

(2) $$Saturated\;density\;(kg/m^{3})=\frac{m2}{m2-m3}\times \rho$$

(3) $$Dry\;density\;(kg/m^{3})=\frac{m1}{m2-m3}\times \rho$$

(4) $$Water\;absorption\;(\%)=\frac{m2-m1}{m1}\times 100$$

where ρ is the density of water at 1000 kg/m^3^ at room temperature. 

### 3.2. PP Volumetric Variation Test

The phase transition and consequent state change of PP is related to the change in its volume. To underline that the purpose of this work was to incorporate liquid PP into recycled aggregates by immersion techniques, it was important to assess the volume changes that PP might suffer and whether this would have a negative impact on recycled aggregates. It is known that the volume change of water in concrete is sufficient to damage it during the freeze–thaw cycle, which is related to the volume expansion of water, which is about 9% [47]. As such, if the volume change associated with the PP phase transition is very high, this may lead to damage to the recycled aggregate, so the PP volumetric variation was measured at controlled temperatures.

**Figure 1:**
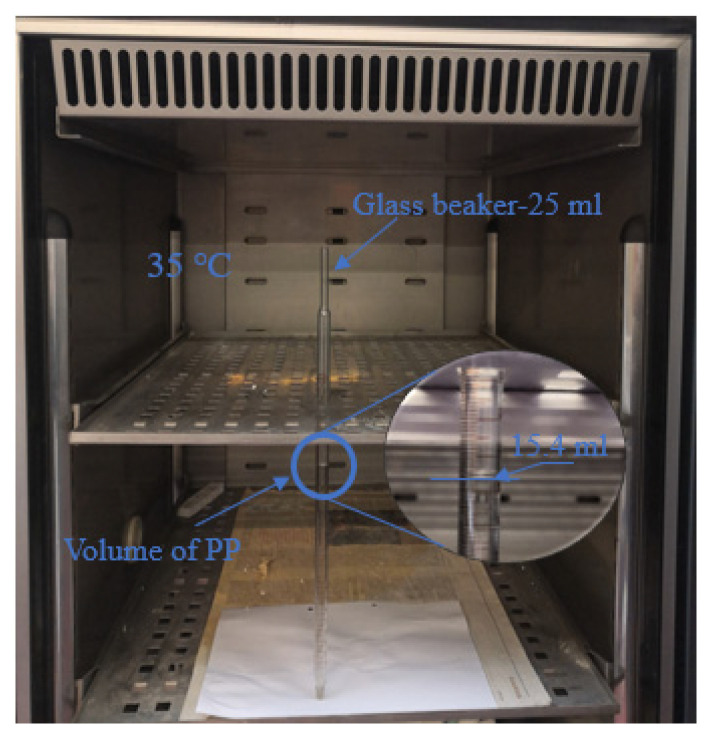
PP volumetric variation test in a temperature-controlled chamber (at 35 °C).

In order to better observe the PP volumetric variation, a long and thin glass beaker with a total volume of 25 mL and a minimum graduation of 0.1 mL was used. The test was carried out in a temperature-controlled chamber. Liquid PP was injected into a beaker at 35 °C (see Figure 1), then the chamber temperature was varied in steps of 5 °C. The test temperature range was 15–35 °C. When the volume change was constant (about 20 min), the volume at this temperature was recorded.

### 3.3. Functionalized Aggregate Production

**Figure 2:**
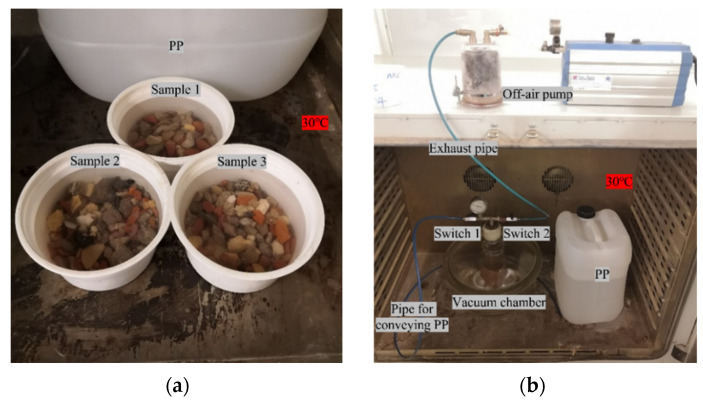
Absorption of RA to PP. (**a**) Absorption at atmospheric pressure; (**b**) absorption under vacuum conditions.

The form-stabilization technique was used for the production of functionalized aggregate with PP. The evaluation of PP absorption by the RA at one and four hours was performed using two distinct methods: absorption at atmospheric pressure (Figure 2a) and absorption under vacuum conditions (Figure 2b). To ensure the optimal absorption of PP by the RA, which had dimensions of 4–10 mm, the RA specimens were pre-dried in an oven at 105 °C for 24 h. In order to assess the RA’s capability to absorb liquid PP, it was essential to maintain the PP in a liquid state throughout the experiments. To achieve this, the PP was placed in an oven at a controlled temperature of 30 °C, which was intentionally higher than the PP’s melting temperature of approximately 22 °C. Subsequently, the RA specimens were immersed in the liquid PP for durations of one and four hours, while being regularly stirred with an iron rod every 15 min to ensure adequate absorption. After one or four hours of absorbing the liquid PP, the RA was extracted using a colander and was then cooled to form RA-PP thermal aggregates. As shown in Figure 2, the surface of the RA-PP aggregate became smooth and moist after the process.

For absorption under vacuum conditions (see Figure 2b), the vacuum chamber was positioned within the oven, while the vacuum pump was situated outside the oven. Initially, RA specimens were placed inside individual boxes, and a total of three specimens were positioned within a vacuum chamber. Subsequently, the port responsible for PP release, which was located on the top of the vacuum chamber, was connected to the RA sample boxes using a catheter. Following the proper sealing of the vacuum chamber, “switch 2” was opened while “switch 1” was closed. The off-air pump was then activated to evacuate the chamber until it achieved a vacuum condition, with the air pressure inside the chamber being monitored through the pressure gauge of the off-air pump. The off-air pump was left running for an additional duration of 3 h to ensure the complete removal of gas from the aggregate pores. Once this step was completed, “switch 2” and the off-air pump were closed. Next, the pipe connected to “switch 1” was immersed in the PP liquid. Upon opening “switch 1”, the PP liquid was released into the RA specimen boxes until the RA specimens were fully submerged. In this state, the absorption of PP for one hour and four hours by the RA specimens were tested, respectively.

### 3.4. Compressive Strength of Concrete with RA-PP

Five concrete mixtures were prepared to observe the effect on the compressive strength of the RA-PP on concrete made in different conditions. The reference concrete (REF) was made with water, cement at 42.5R, river sand with a size of 0–4 mm, and gravel with a size of 4–10 mm. All relationships of water/cement of the concrete mixtures are at 0.575. Then, 40% and 80% samples of RA-PP, made under vacuum conditions (VC) and atmospheric pressure (AP), were substituted for gravel to prepare other mixtures, expressed as 40RA-PP-VC, 40RA-PP-AP, 80RA-PP-VC, and 80RA-PP-AP. The compositions of the concrete mixtures are shown in Table 2.

According to the standard EN 12390-3 (2003) [48], three cylindrical specimens with a dimension of Ø10 cm and 20 cm in height were made. After all specimens were placed in a wet chamber and cured for 28 days, the compressive strength of the concrete was tested.

## 4. Results and Discussions

### 4.1. Physical Properties of RA

#### 4.1.1. RA Composition

**Figure 3:**
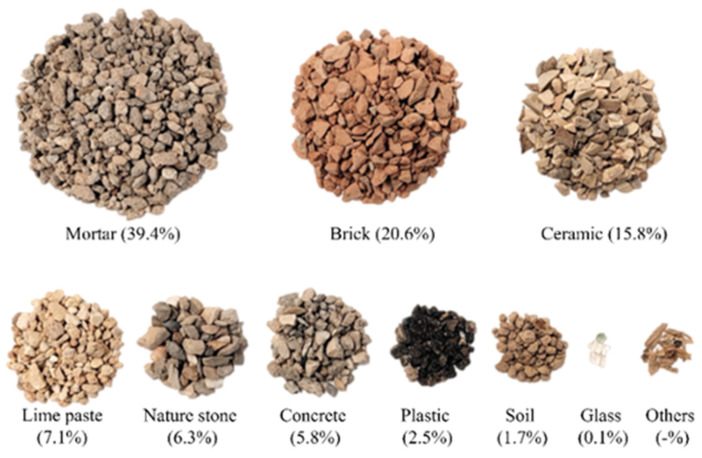
The proportion of each material in the total RA mass.

Through manual sorting, the constituent materials comprising the RA with a dimension of 4–10 mm were obtained, and the proportion of each material within the sample mass was calculated. The results, as shown in Figure 3, revealed the following composition: 39.4% mortar, 20.6% brick, 15.8% ceramic, 7.1% lime paste, 6.3% natural stone, 5.8% concrete, 2.5% plastic, 1.7% soil, 0.1% glass, and traces of other material. These findings align closely with the composition of recycled aggregates produced in the cities of Leiria, Sintra, and Faro in Portugal, as investigated by Bravo et al. [8]. This similarity further indicates that this particular type of recycled aggregate represents the primary recycled aggregate used in Portugal.

#### 4.1.2. Particle Size Distribution of RA

Shown according to the European standard EN 933-1 [45], the results for the rates of materials passing through different sizes of sieves are presented in Table 3. Additionally, the particle size distributions of RA and each constituent material were also examined. It is clear that the size of the largest sieve through which soil and glass passed is 8 mm; lime paste and plastic completely passed through the sieve with a dimension of 10 mm. Other materials were left behind on the sieve with a dimension of 10 mm; specifically, more stones were left on the sieve of 10 mm. This result reveals the constituent materials of RA in the different size ranges and provides further insight into the application of RA.

**Figure 4:**
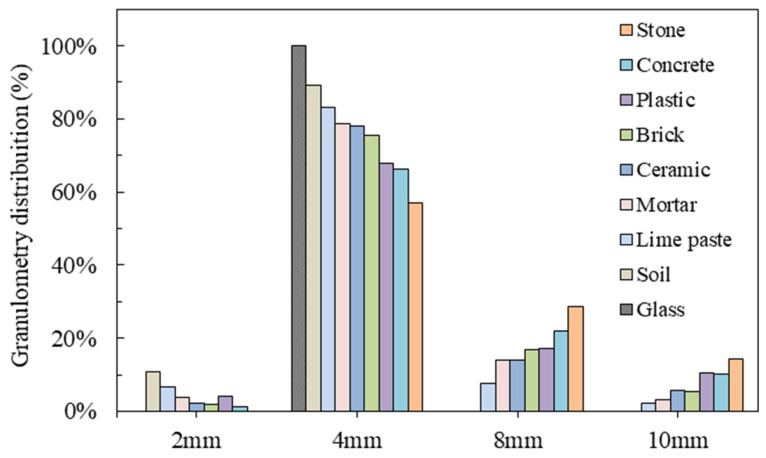
Granulometry distribution of the constituent materials of RA (%).

Figure 4 demonstrates that all the aggregates exhibited a similar trend in terms of particle size distribution. It is evident that the varying proportions of the constituent materials of RA collectively contribute to the overall particle size distribution observed in the RA. The distribution of each type of material at various sizes is clearly depicted in Figure 4. The analysis reveals that a significant portion of the materials exhibited particle size distribution within the range of 4–8 mm, followed by the 8–10 mm range. Notably, the particle size distribution of glass was relatively concentrated, exclusively spanning the 4–8 mm range. On the other hand, concrete, ceramics, mortar, plastic, and brick particles exhibited a relatively uniform distribution, encompassing the entire size range of 4–10 mm. Soil particles, however, were primarily distributed within the 2–4 mm and 4–8 mm ranges.

Upon examining the results, it is apparent that the proportion of each material in different dimension ranges (see Figure 4) and the proportion of each material in the total RA mass (see Figure 3) influence the particle size distribution of the RA (see Table 3). The dominance of particles within the 4–8 mm range suggests the prevalence of specific materials that contribute significantly to this size range. This observation further substantiates the significance of aggregates within the 4–8 mm size range in terms of the absorption of PP. Given their prominent presence and relatively even distribution, the particles within this size range are likely to have a substantial influence on the absorption capacity of the RA. The particle size distribution directly impacts the available surface area and pore structure [49], which are crucial factors governing the absorption behavior. Therefore, understanding the distribution of materials within this size range becomes essential for comprehending the RA’s potential for effectively absorbing PP and informs its future application in various fields.

#### 4.1.3. Density of Recycled Aggregates

In accordance with the NP 581 standard [46], the density of the materials was determined. The results are presented in Table 4. Upon analyzing the outcomes, notable distinctions in the density capacities among the materials become apparent. Stone has the greatest density, followed by concrete, and brick has the least density. The total dry density of the recycled aggregate (RA) mixture was 2143.9 kg/m^3^.

#### 4.1.4. Water Absorption of Recycled Aggregates

In accordance with the NP 581 standard [46], the water absorption of the various materials after 24 h was determined. The results are shown in Table 5.

Of particular interest are the water absorption characteristics, as materials with higher water absorption capacities tend to contribute significantly to the absorption of PP by the RA. From Table 5, it is clear that among the materials tested, brick, lime paste, and mortar exhibit superior water absorption properties (more than the water absorption of RA), suggesting their substantial contribution to the PP absorption capacity by the RA. Conversely, stone, plastic, and glass demonstrate relatively lower water absorption capacities, implying a lesser role in PP absorption.

From the observations in Figure 3, it is evident that mortar, brick, and ceramic collectively account for approximately 75.8% of the total mass of RA. As seen in this study, as a result, the density of these three materials exerts the most substantial influence on the overall density of RA (see Table 4). Furthermore, mortar and brick exhibit significant contributions to enhancing the water absorption capacity of RA. 

From Table 5 and Figure 3, it is evident that the water absorption of RA is related to the water absorption and content of the constituent materials. Among the materials in RA, brick, lime putty, and mortar are higher water-absorption materials that account for about 67% of RA. In contrast, stone, ceramic, and concrete are lower water-absorption materials that account for about 33% of RA. As a result, the RA’s water absorption is inferior to brick, lime putty, and mortar, and is higher for stone, ceramic, and concrete.

In this case, the density and quantity of water absorption of RA can be effectively estimated using the following equation:

Density or quantity of water absorption of RA = Σ Pm × Dm or WAm(5)

where Pm is the percentage of materials (%), Dm is the density of the materials (kg/m^3^), and WAm is the quantity of water absorption of the materials (%).

### 4.2. PP Volumetric Variation

**Figure 5:**
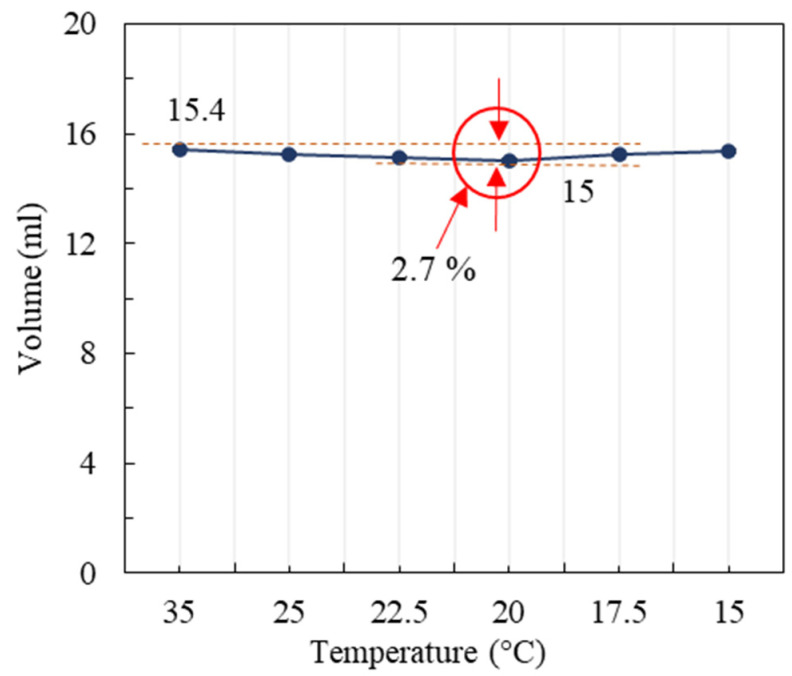
Results of the volumetric variation of PP.

Figure 5 shows the curve representing of the volumetric variation of PP in response to temperature changes. Starting with an initial temperature of 35 °C, a volume of 15.4 mL of PP is injected into the straw. As the temperature progressively decreases, the volume of PP undergoes contraction until it solidifies at 20 °C, resulting in a volume reduction to 15 mL. Subsequently, as the temperature continues to decrease below 20 °C, the volume of PP exhibits expansion, reaching 15.35 mL at 15 °C. The observed data illustrates that the maximum volume change of PP within this temperature range amounts to approximately 2.7% (as shown in Figure 5).

Laboratory observations over a set period of time confirmed that the volume change of PP does not lead to the expansion-related damage of RA-PP, demonstrating the feasibility of using PP in recycled aggregate. This also supports the potential of using PP to improve the thermal performance of RA.

### 4.3. Functionalized Aggregate

**Figure 6:**
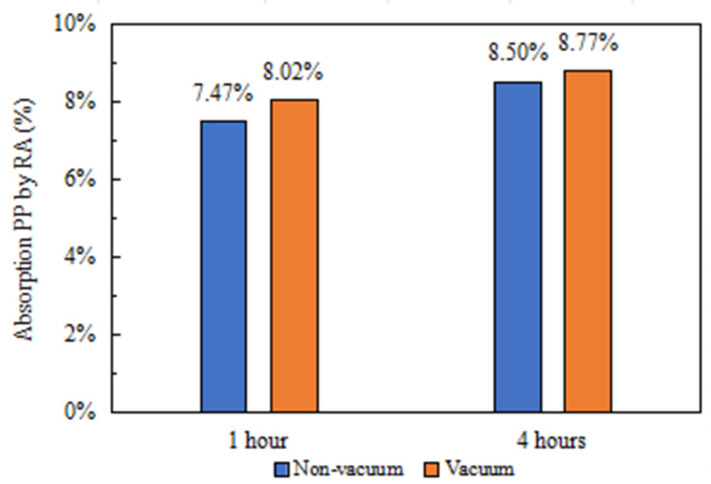
Absorption of PP by the RA.

The study evaluated the absorption of PP by the RA under immersion for one hour and four hours, comparing the absorption under normal atmospheric pressure and vacuum conditions. Figure 6 illustrates the results, showing a 1.03% higher level of absorption of PP by the RA in vacuum conditions compared to that in normal atmospheric pressure after one hour of immersion, and 0.75% higher absorption after four hours of immersion. These findings emphasize the improved efficiency and ease of PP absorption by the RA when the aggregate pores are subjected to vacuum conditions. Additionally, under normal atmospheric pressure, the absorption of PP by the RA increased by 0.55% when the immersion times extended from one to four hours while, under vacuum conditions, the increase was 0.27%. The small difference in PP absorption rate between the vacuum method and the normal atmospheric pressure method after a four-hour absorption period can be explained by the physical properties of the aggregates, which are smaller in size (4–10 mm) and have a larger contact area with liquid PP, making it easier for the liquid to enter the interior of the aggregate, even under normal pressure. Moreover, vacuum adsorption requires power consumption. In conclusion, superior PP absorption by the RA can be achieved under normal atmospheric pressure within four hours.

As shown in Table 4, the dry density of the RA is 2143.9 kg/m^3^. It can be seen that with an immersion time of four hours, RA can absorb 171.9 kg of PP per cubic meter under normal atmospheric pressure conditions and absorb 188 kg of PP per cubic meter under vacuum conditions. Shen et al. [22] demonstrated that CLSC with a dry density of 300–500 kg/m^3^ and a water absorption of 58% can absorb 25% of paraffin, corresponding to an absorption of approximately 75–125 kg of PP per cubic meter. Additionally, Ryms et al. [33] investigated expanded clay aggregates with a density of 1380 kg/m^3^, with dimensions ranging from 4 to 12 mm and a water absorption capacity of 18%, resulting in a PP absorption of 20%. These findings provide evidence that the RA possesses favorable capabilities for PP absorption. Therefore, to maximize the utilization of PP in concrete or other building materials, success can be achieved through the careful adjustment of the proportion of RA in the material mixture.

### 4.4. Compressive Strength of Concrete Mixes

**Figure 7:**
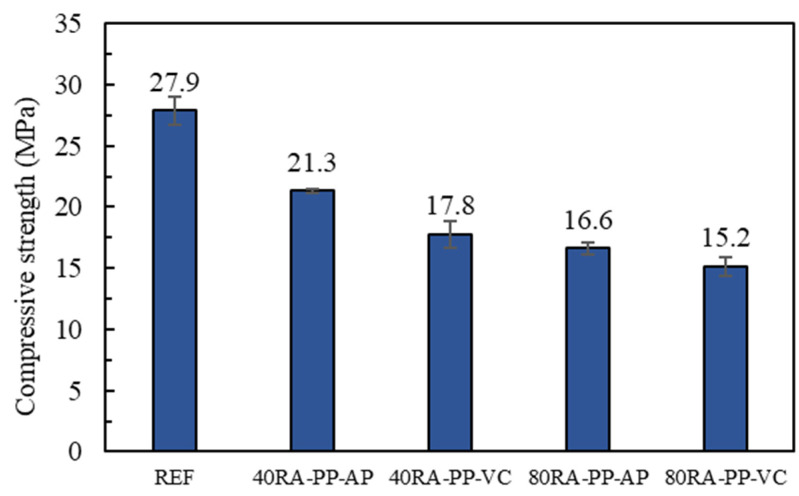
Compressive strength of the various concrete mixes (MPa).

Figure 7 presents the results of the compressive strength tests of concrete mixtures with RA-PP. It is observed that with the content of RA-PP increased, the compressive strength of concrete mixes decreased, for example, in REF, 40RA-PP, and 80RA-PP. The compressive strength of 40RA-PP-AP is 23.6% lower than REF, the compressive strength of 80RA-PP-AP is 22.1% lower than 40RA-PP-AP, and the compressive strength of 80RA-PP-VC is 14.6% lower than 40RA-PP-VC. It was established that the surface of RA-PP is covered with PP, meaning that the increase of RA-PP hinders the contact area between the cement base and the aggregate, thereby reducing the overall compressive strength of the concrete.

Comparing the effect of RA-PP prepared by two methods on the strength of the concrete samples, such as 40RA-PP-VC, 40RA-PP-AP, and 80RA-PP-VC, 80RA-PP-AP and RA-PP, prepared under vacuum conditions, have a stronger degrading effect on the compressive strength of concrete. When the natural aggregate was replaced by 40% of RA-PP, the compressive strength of 40RA-PP-VC decreased by 16.4% compared with that of 40RA-PP-AP, whereas when it was at 80% RA-PP, it decreased by 8.4%. This can be explained by the fact that RA-PP that has been prepared under vacuum conditions contains more PP, and the PP on the aggregate surface would be detached and mixed into the cement slurry during the process of mixing the concrete so the cement slurry will be more porous, further reducing the compressive strength of hardened concrete.

## 5. Conclusions

In response to the global challenges of excessive construction and demolition waste (CDW) generation, along with high building energy consumption, this study explores the integration of phase change technology to enhance the thermal performance of recycled aggregates (RA) as building materials. The investigation focuses on the physical properties of RA for the efficient absorption of phase change materials (PP) to develop a green thermal aggregate, namely, RA-PP.

Physical properties of RA. Particle distribution, density, and water absorption tests were performed on both the RA and its constituent materials. The results reveal that mortar and brick aggregates exhibit the highest capacity for PP absorption by the RA.

The PP volumetric variation test in the phase transition temperature range was carried out in a temperature-controlled chamber. The results show that the maximum volumetric variation of PP during a phase change is 2.7%, which provides support for the feasibility of PP in concrete.

Preparation of RA-PP. The RA-PP samples were prepared under a vacuum and in normal atmospheric pressure conditions. The effects of the two methods on the absorption of PP by the RA at 1 h and 4 h of soaking time, respectively, were analyzed. Interestingly, the PP content of RA-PP prepared using these two methods showed minimal differences (8.02% and 8.77%) when subjected to a four-hour immersion time. From the point of view of complexity and energy consumption, it is recommended that researchers should prepare RA-PP under normal atmospheric pressure for a four-hour immersion time. These findings collectively demonstrate the favorable performance absorption of PP by the RA and the successful development of RA-PP aggregates.

Compressive strength of concrete with RA-PP. The compressive strength of concrete decreased with the increase in RA-PP content. The RA-PP samples prepared under a vacuum had a greater negative impact on the strength of concrete. This further supports that, compared to RA-PP prepared under vacuum conditions, RA-PP prepared under normal atmospheric pressure conditions makes a positive contribution to the strength of concrete.

Overall, this study contributes to addressing the challenges of CDW management and building energy consumption by showcasing the potential of RA as a green material with enhanced thermal performance. The findings highlight the feasibility of using RA-PP aggregates in sustainable building practices and provide data supporting the use of RA-PP in concrete, mortars, and other building materials.

## Figures and Tables

**Table 1:** The characteristics of the PP utilized in this study [44].

Parameters	Values
Density solid (at 20 °C)	0.76 kg/L
Density liquid (at 50 °C)	0.7 kg/L
Heat storage capacity ±7.5%	190 kJ/kg
Specific heat capacity	2 kJ/kg·K
Heat conductivity (both phases)	0.2 W/(m·K)
Flash point	>150 °C
Max. operation temperature	50 °C

**Table 2:** Compositions of the concrete mixes (kg/m^3^).

Mixes	Cement	Water	River Sand 0–4 mm	Gravel 4–10 mm	RA-PP
REF	400	230	393	1035	-
40RA-PP-AP	400	230	393	621	440
40RA-PP-CV	400	230	393	621	446
80RA-PP-AP	400	230	393	207	880
80RA-PP-CV	400	230	393	207	892

**Table 3:** Particle size distribution of each aggregate and RA (%).

Size (mm)	Passing Material (%)									
	Mortar	Brick	Ceramic	Concrete	Lime Paste	Plastic	Glass	Soil	Stone	RA
63	100	100	100	100	100	100	100	100	100	100
31.5	100	100	100	100	100	100	100	100	100	100
16	100	100	100	100	100	100	100	100	100	100
10	99	95	97	95	100	100	100	100	86	99
8	92	78	82	69	88	93	100	100	57	77
4	9	6	4	2	10	7	0	11	0	3
2	0	0	0	0	0	0	0	0	0	0

**Table 4:** Density of the recycled aggregates (kg/m^3^).

	Mortar	Brick	Ceramic	Concrete	Stone	Lime Paste	Plastic	RA
Waterproof material density	2561.2	2620.3	2519.2	2653.6	2661.1	2576.1	2351.0	2575.9
Saturated density	2292.1	2191.4	2402.3	2479.2	2580.3	2279.2	2317.1	2311.5
Dry density	2119.8	1926.7	2325.3	2373.7	2531.6	2090.8	2291.9	2143.9

**Table 5:** Water absorption of the recycled aggregates (%).

	Mortar	Brick	Ceramic	Concrete	Stone	Lime Paste	Plastic	RA
Water absorption	8.13	13.74	3.31	4.44	1.92	9.01	1.10	7.82

## Data Availability

Not applicable.
